# 氨柔比星治疗小细胞肺癌研究进展

**DOI:** 10.3779/j.issn.1009-3419.2010.05.30

**Published:** 2010-05-20

**Authors:** 红阳 卢, 菊芬 蔡

**Affiliations:** 1 310022 杭州，浙江省肿瘤医院肿瘤内科 Department of Medical Oncology, Zhejiang Cancer Hospital, Hangzhou 310022, China; 2 318207 台州，台州市第一人民医院肿瘤内科 Department of Medical Oncology, the First People's Hospital of Taizhou, Taizhou 318020, China

盐酸氨柔比星是日本住友制药株式会社开发全合成的蒽环霉素类抗恶性肿瘤药物，于2002年在日本注册并上市，被批准用于非小细胞肺癌及小细胞肺癌（small cell lung cancer, SCLC）的治疗，本文就氨柔比星治疗小细胞肺癌的研究进展做一综述。

## 1 氨柔比星的分子特征及药代动力学

盐酸氨柔比星（amrubicin hydrochloride）是以9位氨基和糖结构为特征的、化学全合成的新型蒽环霉素类化合物，盐酸氨柔比星及其代谢物氨柔比星醇（amrubicinol）具有DNA内切活性和拓扑异构酶II抑制作用，可使拓扑异构酶II介导的切割复合物稳定，从而切断DNA，生成自由基^[1]^。氨柔比星及其代谢物氨柔比星醇的结构见图 1。氨柔比星的基本药代动力学过程如下。吸收：氨柔比星给药后，原型的血药浓度迅速降低，而其代谢物氨柔比星醇的浓度基本保持平稳，其血浆蛋白结合率约为96%-98%；分布：氨柔比星给药后全身脏器均有药物分布，在骨髓、消化道壁细胞、皮肤、肾、肾上腺、肝脏、脾脏、肺中的药物浓度较高，心脏细胞药物浓度较低；代谢：其主要代谢器官是肝脏，代谢酶主要是羰基还原酶（carbonyl reductase）、NADPH-P450还原酶、NADPH氢-醌还原酶；排泄：主要从胆汁、尿和粪便中排泄；氨柔比星的最大耐受剂量为130 mg/m^2^给药1天、25 mg/m^2^连用5天和50 mg/m^2^连用3天^[2-3]^。

**1:**
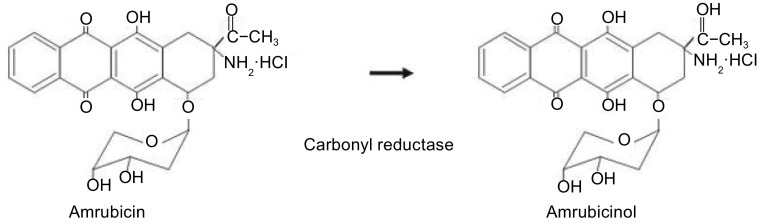
氨柔比星和氨柔比星醇的结构式 The structural formula of Amrubicin and Amrubicinol

## 2 氨柔比星的临床前研究

氨柔比星醇的细胞毒性比氨柔比星强5倍-200倍^[4]^，盐酸氨柔比星体外试验^[5]^显示其细胞增殖抑制作用只有阿霉素（DXR）的1/130-1/2，但氨柔比星醇则显示出与DXR同等或以上的肿瘤抑制作用。Matsunaga等^[6]^研究显示氨柔比星和氨柔比星醇的曲线下面积与其血液学毒性相关。动物实验^[7]^显示氨柔比星仅有很少的迟发性心脏毒性。Hanada等^[8]^进行的体内外研究显示，氨柔比星与顺铂、伊立替康（Irinotecan, CPT-11）、长春瑞宾、吉西他滨、替加氟或曲妥珠单抗联合应用有协同作用，但与吉非替尼联合应用无协同作用。Takigawa等^[9]^研究显示，对CPT-11的活性代谢产物SN-38耐药的SBC-3/SN-38细胞对氨柔比星敏感，且其与顺铂有协同或相加作用；且对顺铂耐药的SBC-3/CDDP细胞对氨柔比星也仍然敏感，其与CPT-11也具有协同或相加作用。此研究为氨柔比星用于SCLC的二线治疗提供了理论支持。

## 3 氨柔比星一线治疗SCLC

### 3.1 氨柔比星单药

Yana等^[10]^进行了氨柔比星治疗初治的广泛期SCLC的Ⅱ期临床研究，主要目的是评价其疗效和安全性，共35例患者入组，33例合格患者可评价疗效和安全性。氨柔比星45 mg/m^2^第1、2、3天，三周重复，如果治疗的第一周期结束后出现肿瘤增大25%或第二周期后出现肿瘤增大50%则进行挽救性化疗。结果显示3例完全缓解（complete response, CR），22例部分缓解（partial response, PR），中位生存期为11.7个月。主要的毒性是血液学毒性，3级以上的非血液学毒性为：厌食（9.1%）和脱发（3.0%）。6例患者进行挽救性化疗。具体见表 1。Yana等^[10]^认为氨柔比星对于广泛期SCLC是有效的且其毒性可以接受，其联合化疗值得进一步研究。

**1:** 氨柔比星治疗一线治疗SCLC临床研究^[3]^ Studies of amubicin in first-line treatment

Author	Type of study	No. of patients	Overall response	Median survival/month
Yana T, *et al*^[10]^	Single arm	35	78.8%	11.3
Ohe Y, *et al*^[11]^	Single arm	44	87.8%	13.6

### 3.2 氨柔比星联合顺铂

顺铂是SCLC一线治疗的基础用药，Ohe等^[11]^进行了氨柔比星联合顺铂治疗初治的广泛期SCLC的研究，研究显示其最大耐受剂量为氨柔比星45 mg/m^2^第1、2、3天，顺铂60 mg/m^2^第1天。另有41例给予推荐用量（氨柔比星40 mg/m^2^第1、2、3天，顺铂60 mg/m^2^第1天），有4例CR，32例PR，1年生存率为56.1%，中位生存期为13.6个月。其3级以上的血液毒性为血红蛋白减少（53.7%）、白细胞减少（65.9%）、中性粒细胞减少（95.1%）、血小板减少（24.4%）。氨柔比星与顺铂联合应用的骨髓抑制较严重，但可以通过给予粒细胞集落刺激因子（granulocyte colony-stimulating factor, G-CSF）得到控制。具体见表 1。此研究为开展氨柔比星联合顺铂治疗初治的广泛期SCLC奠定了良好的基础，国内现正在进行盐酸氨柔比星联合顺铂化疗与依托泊苷联合顺铂化疗对照治疗广泛期SCLC的Ⅲ期临床试验（方案编号：D0750018），我院作为该临床试验参加单位之一，已经有患者入组，在氨柔比星联合顺铂组骨髓抑制较明显，在实际应用中需密切注意3级以上的中性粒细胞减少的发生。

### 3.3 氨柔比星联合CPT-11

Oshita等^[12]^进行了氨柔比星联合CPT-11治疗初治的广泛期SCLC的研究，共13例患者入组。其最大耐受剂量为氨柔比星40 mg/m^2^第1、2、3天，CPT-11 60 mg/m^2^第1天，并在第5天-第9天预防性应用G-CSF支持，在此剂量组中的4例患者中有3例患者出现4度中性粒细胞减少性发热。第2剂量组为氨柔比星35 mg/m^2^第1、2、3天，CPT-11 60 mg/m^2^第1天，也同样在第5天-第9天预防性G-CSF支持，在6例患者中仅1例患者出现4度中性粒细胞减少。总的研究结果显示13例患者中1例CR，12例PR，1年生存率为76.9%，中位生存期为17.4个月。该作者认为G-CSF支持下氨柔比星联合CPT-11治疗初治的广泛期SCLC的推荐用量为氨柔比星35 mg/m^2^第1、2、3天，CPT-11 60 mg/m^2^第1天。此研究说明氨柔比星与CPT-11有较好的协同作用，其联合应用是安全而有效的，值得进一步的研究。

### 3.4 氨柔比星联合卡铂

卡铂也是SCLC一线治疗的重要药物，特别是对老年患者耐受性明显好于顺铂。Fukuda等^[13]^进行了氨柔比星联合卡铂一线治疗SCLC的Ⅰ期临床研究，16例患者入组，可评价病例为15例，分3个剂量水平，分别为氨柔比星40 mg/m^2^、35 mg/m^2^和30 mg/m^2^，均为第1、2、3天使用，卡铂均按AUC为5计算，第1天使用。其中2例CR，9例PR，3例稳定（stable disease, SD），1例进展（progressive disease, PD），中位生存期为13.6个月。最大的耐受剂量为氨柔比星40 mg/m^2^第1、2、3天，卡铂按AUC为5计算，第1天使用，推荐剂量为氨柔比星35 mg/m^2^第1、2、3天，卡铂按AUC为5计算，第1天使用。此研究为氨柔比星联合卡铂治疗SCLC奠定了基础，可作为以后研究的重要参考。Inoue等^[14]^进行了氨柔比星联合卡铂治疗初治的老年SCLC的Ⅰ期研究，主要是为了观察剂量限制性毒性和最大耐受剂量，拟分3个剂量组进行，共12例患者入组。第1剂量组为氨柔比星40 mg/m^2^第1、2、3天，卡铂按AUC为4计算，第1天使用，三周重复，结果3例患者均出现剂量限制性毒性（持续4天以上的4度中性粒细胞减少，血小板低于20 000/mm^3^，或3度腹泻）。另外9例患者均降低一个剂量水平进行，氨柔比星35 mg/m^2^第1、2、3天，卡铂按AUC为4计算，第1天使用，三周重复，9例患者中3例出现了剂量限制性毒性，但这些毒性不是非常严重而且是可控的，非血液学毒性是轻微、可逆的。总的客观有效率为83%，中位生存期为12.7个月。因此认为氨柔比星联合卡铂治疗初治的老年SCLC的最大耐受剂量为氨柔比星40 mg/m^2^第1、2、3天，卡铂按AUC为4计算，第1天使用，三周重复。推荐剂量为氨柔比星35 mg/m^2^第1、2、3天，卡铂按AUC为4计算，第1天使用，三周重复。此Ⅰ期研究为以后的Ⅱ期临床研究提供了依据，并摸索出了氨柔比星联合卡铂治疗初治的老年SCLC的推荐用量。随后Inoue等^[15]^进行了氨柔比星联合卡铂一线治疗老年SCLC的Ⅱ期临床研究，氨柔比星35 mg/m^2^第1、2、3天，卡铂按AUC为4计算，第1天使用，36例患者入组，总有效率为89%，中位生存期为18.6个月，其3级及以上的中性粒细胞下降为97%，3级及以上的中性粒细胞减少性发热为17%，无治疗相关性死亡的发生。因此认为氨柔比星联合卡铂一线治疗老年SCLC是相当有效的，其毒性是可以接受的，值得进一步研究。但国内卢红阳等^[16]^认为氨柔比星联合卡铂一线治疗老年SCLC的疗效尚可，但中性粒细胞减少发生率太高，不主张常规应用。我们在实际应用中应该高度重视可能出现的骨髓抑制。

## 4 氨柔比星治疗复治的SCL

### 4.1 氨柔比星二线或三线治疗的推荐剂量

Igawa等^[17]^进行了氨柔比星二线或三线治疗SCLC的临床研究，结果显示氨柔比星40 mg/m^2^第1、2、3天给药对于二线治疗来说其毒性是可以耐受的，但对于三线治疗的7例患者中3例出现了3级非血液学毒性，4例患者出现了4级中性粒细胞下降，因此认为此剂量对于三线治疗来说其毒性是不能耐受的。随后用氨柔比星35 mg/m^2^第1、2、3天三线治疗了7例SCLC患者，仅1例患者出现了中性粒细胞减少性发热，1例患者出现了4级中性粒细胞下降，认为其毒性是可以接受的，同时也具有一定的疗效，其中1例PR，2例SD。此研究为氨柔比星二线或三线治疗SCLC所需的安全而有效的剂量提供了重要的参考。Kaira等^[18]^进行了氨柔比星二线治疗肺癌的Ⅱ期临床试验，在SCLC组氨柔比星用法为35 mg/m^2^第1、2、3天，三周重复，共29例SCLC患者入组，其3级以上中性粒细胞减少的发生率为41.4%，无治疗相关性的死亡，有效率为44.8%，生存期为12.0个月，1年生存率为46.7%。Kaira等^[18]^认为氨柔比星二线SCLC是有效的且有较好耐受性，氨柔比星用法为35 mg/m^2^，与40 mg/m^2^相比有相似的疗效和更少的毒性，值得进一步研究。

### 4.2 氨柔比星二线治疗的疗效

Onoda等^[19]^进行了氨柔比星治疗SCLC的Ⅱ期临床研究，共有60例患者入组，敏感复发组44例，难治性组16例。所有患者均曾行至少一个含铂方案化疗，患者ECOG评分为0-2分，氨柔比星40 mg/m^2^第1、2、3天，21天为一个周期，中位治疗周期数为4个周期。敏感复发组的有效率为52%，难治性组的有效率为50%，敏感复发组的中位生存期为11.6个月，难治性组为10.3个月。总的3级及以上中性粒细胞减少的发生率为83%，血小板减少为20%，贫血为33%，中性粒细胞减少性发热为5%，非血液学毒性是轻微的。具体见表 2和表 3。Onoda等^[19]^认为氨柔比星治疗SCLC有明显疗效，其毒性是可以预测和控制的。Ettinger等^[20]^进行了氨柔比星二线治疗难治性的且经铂类一线化疗的广泛期SCLC的Ⅱ期临床试验，共有75例患者入组，氨柔比星40 mg/m^2^第1、2、3天，21天为一个周期，直至疾病进展或不能耐受毒性，69例患者完成中位4个周期的化疗，6例患者死亡或未行化疗，总有效率为21%（其中1例CR），中位生存期为6个月，3级及以上中性粒细胞下降、血小板下降和白细胞下降分别为65%、39%和35%，中性粒细胞减少性发热为10%，38%的患者进行了剂量调整。具体见表 3。因此认为氨柔比星二线治疗难治性的且经铂类一线化疗的广泛期SCLC有较好的疗效和可接受的安全性。以上的研究显示氨柔比星二线治疗难治性的或敏感复发的SCLC均有较好的疗效，值得进一步的对照研究。

**2:** 氨柔比星治疗敏感复发或难治性SCLC Ⅱ期临床研究结果 Phase Ⅱ study of amrubicin in relapsed case or refractory case with small lung cancer: response

	Sensitive cases (*n*)	Refractory cases (*n*)	Total (*n*)
No. of patients	44	16	60
CR	1	1	2
PR	22	7	29
SD	10	2	12
PD	11	6	17
Response rate (95%CI)	52% (37%-68%)	50% (25%-75%)	52% (38%-65%)
Progression-free survival (95%CI)/month	4.2 (3.6-5.3)	2.9 (1.4-4.6)	3.9 (3.4-4.6)
Median survival time (95%CI)/month	11.6 (10.0-15.8)	10.3 (4.8-∞)	11 (10.0-13.2)
1-year survival (95%CI)	45.5% (29.9-59.8)	40.3% (15.1-64.6)	44.1% (30.6-56.8)
∞:a symbol of infinite. CR: complete response; PR: partial response; SD: stable disease; PD: progressive disease.			

**3:** 氨柔比星二线治疗SCLC临床研究^[3]^ Studies of Amubicin in second line setting

Author	Type of study	Patient population	No. of patients	Overall response	Median progression free surviva/month	Median survival/month
Onoda S, *et al*^[19]^	Single Arm	Sensitive	40	52%	4.2	11.6
		Refractory	16	50%	2.6	10.3
Ettinger D, *et al*^[20]^	Single Arm	Refractory	75	17.2%	3.2	----
Jotte RM, *et al*^[22]^	Randomized phase Ⅱ	Sensitive				
		Amrubicin	50	34.7%	4.6	----
		Topotecan	26	3.8%	3.5	----
Inoue A, *et al*^[21]^	Randomized phase Ⅱ	Sensitive				
		Amrubicin	17	53%	3.9	9.9
		Topotecan	19	21%	3.0	11.7
		Refractory				
		Amrubicin	12	17%	2.6	5.3
		Topotecan	11	0%	1.5	5.4

### 4.3 氨柔比星与拓泊替康对照研究

拓泊替康是美国食品药品监督管理局批准用于SCLC二线治疗的药物。Jotte等^[21]^进行了氨柔比星与拓泊替康二线对照治疗广泛期SCLC的Ⅱ期临床试验，氨柔比星40 mg/m^2^第1、2、3天，拓泊替康1.5 mg/m^2^第1、2、3、4、5天，结果显示氨柔比星的疗效优于拓泊替康，且氨柔比星组未发现心脏毒性。Jotte等^[22]^进行了氨柔比星与拓泊替康对照二线治疗对铂类一线化疗敏感的广泛期SCLC的Ⅱ期临床试验，氨柔比星40 mg/m^2^第1、2、3天，拓泊替康1.5 mg/m^2^第1、2、3、4、5天，21天为一个周期，氨柔比星组50例，中位治疗周期数为6个周期，拓泊替康组26例，中位治疗周期数为3个周期，氨柔比星组比拓泊替康组有更高的有效率（36% *vs* 8%, *P* < 0.012），氨柔比星组中位生存期为9.3个月，拓泊替康组中位生存期为8.9个月，ECOG评分为2分的患者中氨柔比星组有6例，拓泊替康组2例。在ECOG评分为0分-1分的患者中，氨柔比星组有提高生存期的趋势，氨柔比星组的中位生存期为10.5个月，拓泊替康组为9.7个月，3级的中性粒细胞减少的发生率分别为53%和74%，3级血小板减少分别为31%和52%，3级白细胞减少为27%和30%，另外氨柔比星组中的3例（6%）和拓泊替康组中的1例患者（4%）死于感染。具体见表 3。因此认为与拓泊替康相比，对于铂类一线化疗敏感的广泛期SCLC，氨柔比星二线治疗提高了有效率，且毒性可以耐受。Inoue等^[23]^进行了氨柔比星与拓泊替康对照治疗复治的SCLC的Ⅱ临床研究，随机分为两组，氨柔比星组为40 mg/m^2^第1、2、3天，拓泊替康组为1.0 mg/m^2^第1、2、3、4、5天，均为21天为一个周期，共60例患者入组，可评价病例为59例，敏感复发患者36例，难治性复发患者23例，氨柔比星组1例患者因感染而出现治疗相关性死亡。在敏感复发患者中氨柔比星组的有效率为53%，拓泊替康组有效率为21%。难治性复发患者中氨柔比星组的有效率为17%，拓泊替康组有效率为0%。氨柔比星组的中位无进展生存期为3.5个月，而拓泊替康组为2.2个月。具体见表 3。因此认为氨柔比星治疗复治的SCLC优于拓泊替康，并且其毒性可以接受的。

### 4.4 二线治疗小结

上述的临床研究显示氨柔比星对于SCLC的二线治疗有较好的疗效，其疗效优于拓泊替康，氨柔比星二线治疗SCLC的Ⅲ期临床试验也正在进行中^[3]^，我们将期待其最终结果的发布。在SCLC二线治疗中氨柔比星的合适剂量可为35 mg/m^2^第1、2、3天，三周重复。目前关于SCLC的二线化疗2010年美国国家癌症综合治疗联盟（NCCN）的推荐为：参加临床试验；2个月-3个月内复发且PS评分为0分-2分的患者可以考虑应用的药物为异环磷酰胺、紫杉醇、多西紫杉醇、吉西他宾、伊立替康、托泊替康；2个月-3个月后至6个月内复发的可以考虑应用托泊替康（1级证据）、CAV、吉西他宾、紫杉醇、多西紫杉醇、口服依托泊甙和长春瑞宾；6个月后复发的可以考虑原方案。在实际选用二线治疗方案时应充分考虑患者的全身情况、一线化疗的情况以及所选药物的毒副作用等。

## 5 安全性

参考文献Shimokawa等^[24]^回顾性分析氨柔比星治疗难治性或复发的SCLC，认为严重的骨髓抑制影响了其安全性。氨柔比星无论是单药还是联合化疗，不管是一线应用还是二线应用，均会产生较为严重的骨髓抑制作用，故需要高度重视。Goto等^[25]^报道了1例应用氨柔比星治疗正在进行血液透析的肾衰竭患者，患者年龄为83岁，有肝转移，使用2周期氨柔比星后疗效达PR，毒性是轻微的，检测发现患者在透析期间与非透析期间血中氨柔比星和氨柔比星醇的浓度无差别。因此认为氨柔比星可能是正在进行血液透析的肾衰竭SCLC患者的一个较好选择。Jotte等^[21]^的研究显示氨柔比星有较好的心脏安全性，这一点明显优于其它蒽环霉素类化合物。

## 6 小结

氨柔比星在SCLC的一线治疗中有较好的疗效，氨柔比星联合顺铂治疗初治的广泛期SCLC的中位生存期^[11]^超过了日本JCOG 9511研究^[26]^中报道的IP方案组12.8个月的中位生存期。氨柔比星联合卡铂治疗初治的老年SCLC同样也具有较好的疗效，推荐剂量可为氨柔比星35 mg/m^2^第1、2、3天，卡铂按AUC为4计算，第1天使用，三周重复。氨柔比星二线治疗难治性或敏感复发的SCLC均有较好的疗效，Ⅱ期临床试验显示疗效优于拓泊替康。欧美国家也正在进行氨柔比星治疗SCLC的临床试验^[3, 27]^，国内外氨柔比星一线或二线治疗SCLC的Ⅲ期临床试验正在进行中^[3]^，我们将期待其最终结果的报道。氨柔比星有较好的心脏安全性，对肾功能欠佳患者可能也是一个较好的选择，其主要毒副作用为血液学毒性，需要我们高度重视。
